# Challenging dilemmas of low grade, non-invasive bladder cancer: a narrative review

**DOI:** 10.1590/S1677-5538.IBJU.2021.0259

**Published:** 2021-06-20

**Authors:** Fernando Korkes, Phillipe E. Spiess, Herney Andres Garcia-Perdomo, Andrea Necchi

**Affiliations:** 1 Faculdade de Medicina do ABC Disciplina de Urologia Santo Andre SP Brasil Disciplina de Urologia, Faculdade de Medicina do ABC - FMABC, Santo Andre, SP, Brasil; 2 Hospital Israelita Albert Einstein Disciplina de Urologia São Paulo SP Brasil Disciplina de Urologia do Hospital Israelita Albert Einstein, São Paulo, SP, Brasil; 3 H. Lee Moffitt Cancer Center and Research Institute Department of Genito-Urinary Oncology Tampa FL EUA Department of Genito-Urinary Oncology, H. Lee Moffitt Cancer Center and Research Institute, Tampa, FL, EUA; 4 University of South Florida University of South Florida Department of Urology and Oncology Tampa FL EUA Department of Urology and Oncology, University of South Florida University of South Florida, Tampa, FL, EUA; 5 Universidad Del Valle School of Medicine Department of Surgery Cali Colômbia Division of Urology / Urooncology, Department of Surgery, School of Medicine, Research Group - UROGIV, Universidad Del Valle, Cali, Colômbia; 6 University Vita-Salute San Raffaele Discipline of Urology Milan Italy Discipline of Urology, University Vita-Salute San Raffaele, Milan, Italy

**Keywords:** Urinary Bladder Neoplasms, Carcinoma, Transitional Cell, Review Literature as Topic

## Abstract

**Purpose:**

To describe the current scientific knowledge and clinical experience in low-grade-non-muscle-invasive bladder cancer (LG-NMIBC) patients in challenging scenarios.

**Materials and Methods:**

Medline, Embase, Google Scholar, and Cochrane Central were searched until March 2021.

**Results:**

A total of 841 studies were identified, and abstracts were analyzed. Twenty-one relevant studies were then identified and reviewed. After all, information was gathered from 16 studies, the authors discussed the specific topics, and expert opinions were also included in the discussion. There have been some studies that can help us to have some insights on how to manage these patients. Very distinctive strategies have been reported in the literature, mainly anecdotally or in small randomized studies. Some of these treatments outlined in the present manuscript include repeated TURBTs, chemoablation, BCG immunoablation, partial cystectomy, radical cystectomy, radiotherapy, chemotherapy, and future perspectives. In the current manuscript, we have combined these strategies in a proposed algorithm.

**Conclusion:**

For those LG-NMIBC patients in challenging scenarios, we have found repeated TURBTs, chemoablation, BCG immunoablation, partial cystectomy, radical cystectomy, radiotherapy, and chemotherapy are attractive modalities to treat them effectively. Also, the current manuscript proposes an algorithm to overcome these challenges.

## INTRODUCTION

Bladder cancer is the sixth most common cancer in the US and represents 4.6% of all new cancer diagnoses, equivalent to 80.470 new cases and 17.670 deaths in the US during 2019 (1). It also has significantly elevated expenses and perhaps the highest lifetime treatment costs per patient (2).

In high-risk non-muscle-invasive bladder tumors (NMIBC), radical progression and metastasis are significant concerns. The standard treatment of these patients is TURBT and BCG installations. Nonetheless, a radical cystectomy is a good option (3). On the other side, for muscle-invasive bladder cancer (MIBC), the last one is the standard intervention. However, nowadays, there is increasing evidence that trimodal therapy (complete TURBT, chemotherapy, and radiotherapy) might be an essential and acceptable intervention for selected cases (low-volume T2, absence of CIS, no hydronephrosis) (4).

Non-muscle invasive bladder cancer (NMIBC) is commonly treated and cured through transurethral resection of the bladder tumor (TURBT). Low-grade, non-invasive tumors rarely metastasize, the high recurrence rates and progression risk are avoided through adjuvant measures and an extensive follow-up program (5). Even though TURBT is a standard procedure mastered by most urologists, there are certain challenging situations. Sometimes the urologist faces a TURBT with an NMIBC located in an inaccessible position, a large prostate / urethral stricture precluding the resectoscope introduction or an extensive low-grade Ta lesion that cannot be endoscopically resected. Accordingly, large-volume, multifocal cancers can usually be managed with conservative techniques with a good prognosis (6).

We aimed to describe the current scientific knowledge and clinical experience LG-NMIBC patients challenging scenarios. An international panel of experts on bladder cancer treatment performed a review and identified alternatives in complex TURBT cases for LG NMIBC.

## MATERIALS AND METHODS

We conducted this comprehensive review following Joanna Briggs Institute recommendations (7).

**Eligibility criteria:** Studies including alternative interventions for patients over 18 years of age with a Ta NMIBC diagnosis and considered complex TURBT.

**Information sources:** We carried out the literature search in the MEDLINE (OVID), EMBASE, Google Scholar, and CENTRAL databases from inception to March 2021. We performed a structured search using terms and synonyms related to the condition of interest.

## DATA COLLECTION

Two researchers identified each reference by title and abstract. Subsequently, we reviewed the full texts of relevant studies and applied pre-specified inclusion and exclusion criteria. Using a standardized form, the reviewers independently extracted the following information from each article.

Data synthesis: We showed each clinical trial result descriptively, trying to respond to the proposed objective.

## RESULTS

### Study selection

**Figure 1:**
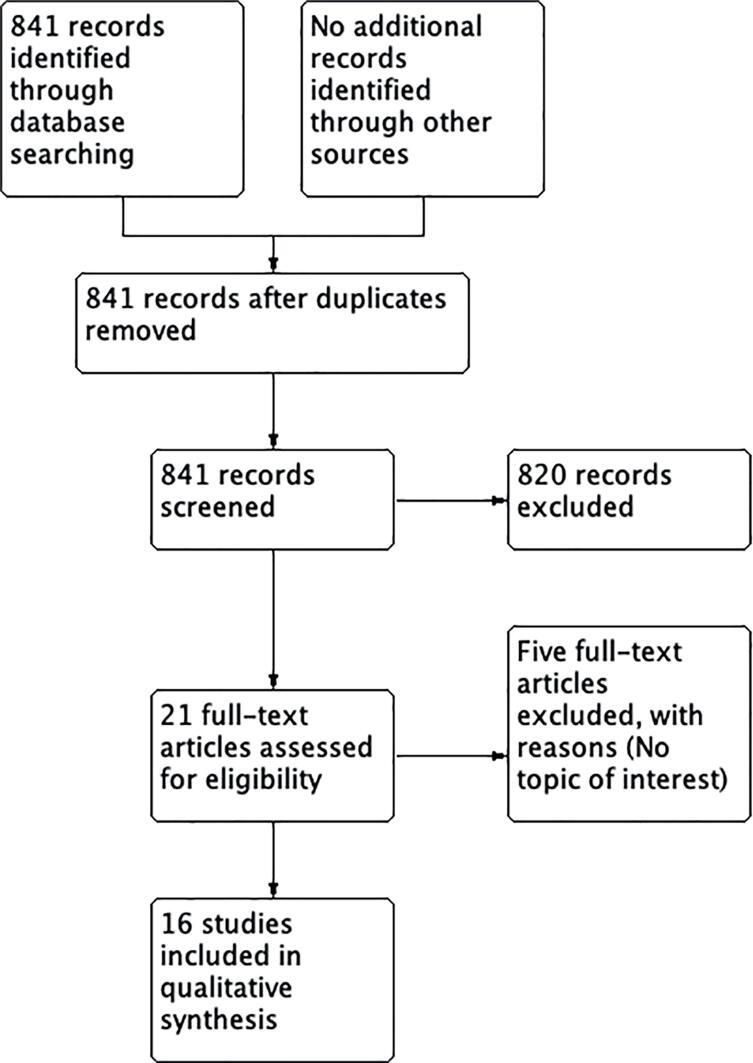
Flowchart for study selection.

We identified 841documents from the search strategy. Finally, we included sixteen studies that were eligible for our review. (Figure-1)

### Characteristics of included studies

We found multiple design studies, including primary studies, reviews, and commentaries. They were all published in worldwide journals as the primary purpose of this study is to present the strategies, as a way to standardize alternative interventions in complex cases, we go ahead to present them.

### Specific strategies

#### Strategies to access the tumor

##### Difficult-to-reach tumors

At times, urologists have to deal with challenging situations at the TURBT, such as large prostates, large-distended bladders, severe urethral strictures or stenosis, and often obese patients making access to the bladder tumor foci quite prohibitive (Table-1). Additionally, tumors located at the bladder dome and anterior bladder wall can pose additional difficulties. In these cases, conventional maneuvers as emptying the bladder, suprapubic pressure, or Trendelenburg position are not helpful tips.

**Table 1:** Frequent situations associated with LG-NMIBC challenges.

Challenging situations to treat LG-Ta NMIBC*:
1 - Large prostates
1 - Obese patients
2 - Large distended Bladder (not highly compressible)
3 - Severe urethral stricture(s) or stenosis/small urethral caliber
4 - Difficult location (inaccessible bladder dome/anterior bladder wall)
5 - Difficult location (bladder neck)
6 - Extensive LG-Ta (probably the most frequent scenario)

* low grade non muscle-invasive bladder cancer (LG-Ta NMIBC)

Correspondingly, the cystoscope or even the ureteroscope might be valuable tools to perform this procedure. It is possible to perform a cold-cup biopsy and Bugbee cauterization of some LG lesions through the cystoscope. Also, laser ablation or en-bloc resection with the resecoscope might be possible and accessible.

An extra-long resectoscope may be another tool to use in these challenging situations. If not readily available, the procedure can be postponed. Also, an old but exciting technique is a perineal urethrostomy, which can also be used as an access route in challenging cases (8, 9).

##### Extensive tumors

In multiple LG-NMIBC tumors with almost no normal urothelium, the surgical resection might be difficult and dangerous. It is crucial to ensure a good visualization throughout the procedure, controlling significant hematuria, cauterizing bleeders throughout the resection, and evacuating clots with an Ellik or some other means of effective evacuation. For extensive tumors, incomplete resection may be unavoidable sometimes, and staged procedures are the safest approach. Even though there are no current formal recommendations for such cases, adjuvant treatment strategies, as mentioned below, can be of value.

#### Treatment strategies

##### Staged procedures

For extensive tumors, incomplete resection may be unavoidable sometimes, and staged procedures can be the safest approach (6). In such cases, we suggest a complete tumor resection in one area, providing meticulous hemostasis. A second procedure can be scheduled between two to four weeks to complete the procedure.

Also, for patients with a huge prostate or a huge median lobe that precludes access to a bladder tumor, benign prostatic hyperplasia can be initially treated, and the TURBT can be performed as a staged procedure.

Alternatively, if there is a high-volume prostate, it can be resected as a first-step procedure, and then a delayed TURBT in 6-8 weeks.

##### Chemoablation of NMIBC

A few studies describing chemoresection or chemoablation as an alternative to TURBT have been published during the last decade. Bono et al. (10) evaluated mitomycin C (MMC) and epirubicin in two EORTC trials. They observed 57% and 67% complete response rates, respectively.

Similarly, in a prospective trial, Lindgren et al. (11) treated 120 patients with Ta-NMIBC (LG or HG), with intravesical MMC with 40mg/40mL/2 hours, three times a week for two weeks. They found 57% complete tumor response at four weeks. Interestingly, adverse events were less common after chemoablation than after TURBT plus MMC or BCG.

Colombo et al. (12) included 54 small-LG-Ta NMIBC patients. Patients received a weekly MMC instillation/6 weeks or three instillations/week for two weeks. They found a 70.4% complete response after 14 days. Contrarily, Mostafied et al. (13) evaluated 82 small LG-Ta-NMIBC patients. They only found 37% complete responses after four MMC instillations for one week.

It seems that a more intense (3x / week) and more extended period (two weeks) chemoablation with MMC might be more effective. Nonetheless, this is low-quality evidence, and we need high-quality clinical trials for decision-making. Other chemotherapies have not been tested in this setting.

Gemcitabine has also been studied in incomplete resection settings (14). A 6x / weekly gemcitabine reached a 23% complete response. An escalated dose of 2.000mg achieved a better complete response (33.3%). Another study found a similar 31% complete response in this setting (15).

##### BCG ablation

BCG is currently recommended as an adjuvant measure to reduce NMIBC recurrence after TURBT in high-risk patients. Nonetheless, it has been tested as a neoadjuvant treatment strategy in only one study (16) (They also previously reported their outcomes with almost the same results (17)). Akaza et al. applied 80mg weekly BCG for eight weeks in 125 Ta, T1, or CIS patients before TURBT. There was a complete response in 66.4% of the papillary tumors. For Cis, there was an 84% complete response (16). It is noteworthy that this approach has been tested in a very controlled trial setting and needs more evidence to extrapolate its results. For larger tumors, persistent hematuria might delay treatment and require emergency treatments. It should therefore be considered very cautiously. To our knowledge, there are no other studies for this intervention.

##### Partial Cystectomy

Partial cystectomy (PC) is considered a treatment only for exceptional cases of urothelial bladder carcinoma. Even though there is no consensus regarding this intervention, the main indications are single tumors in diverticula or T2-small-single tumor with good bladder capacity in a favorable position and without extensive CIS.

One of the most extensive available series about this intervention in this setting is published by Capitanio et al. (18). They analyzed the SEER database and observed that 23.3% of all 1.753 PC were performed for Ta tumors. There was no recurrence nor other oncological outcomes report.

**Figure 2:**
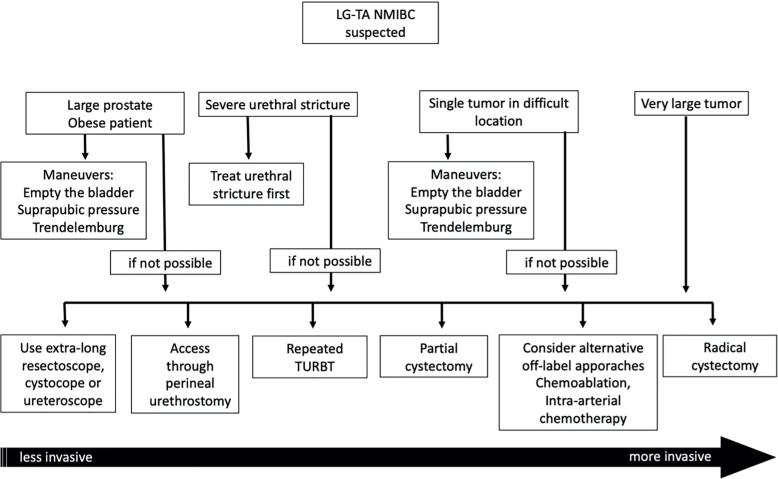
Algorithm for challenging LG-NMIBC management.

This situation is not widely mentioned in the literature and guidelines; however, there might be some room for PC in NMIBC. For instance, single large LG/Ta tumors, close to the bladder neck and not easily accessible by TURBT (Figures 2a and 2b).

##### Radiotherapy

Urothelial carcinoma is a radio-sensitive tumor. Radiotherapy may not be considered as monotherapy for treating MIBC. Instead, combined with chemotherapy and TURBT (Trimodal therapy) has essential effects in oncological outcomes of selected patients, even with fiducial markers as new tools for improving effectiveness (4, 19).

Rodel and Akcetin tried radiotherapy and radiochemotherapy in high-risk T1 bladder cancer. They found an 83-90% complete remission after TURBT. Also, overall survival of 75% at five years and 50% at ten years (20, 21).

Weiss et al. reported radiotherapy or chemoradiotherapy as an alternative for high-risk T1 bladder cancer (22). They found 88% complete response, 30% progression at ten years, and disease-specific survival of 73% at ten years. However, there are no high-quality studies to confirm this data. We did not find any information for low-grade or large volume Ta tumors.

##### Neoadjuvant arterial chemotherapy

We found a single report of such treatment for an extensively large papillary NMIBC patient who was not amenable to endoscopic resection. This 50-year-old man underwent an arterial infusion of cisplatin (100mg/body) into the superior vesical artery twice, with a 5-week interval. A ten-time fold reduction in tumor volume was observed, and the low-grade-Ta tumor was rendered amenable to TURBT (23).

This use of single-agent intra-arterial chemotherapy seems to be an exciting strategy to be considered in low-grade large-volume NMIBC, where bladder preservation is intended.

##### Radical Cystectomy

Radical Cystectomy (RC) is currently considered the gold-standard treatment for patients with MIBC (24). In NMIBC patients, RC is an option, mainly considered after BCG failure, especially for high-risk or very high-risk patients, unreachable T1 tumors, residual T1 tumors after resection, or high-grade tumors with CIS and lymphovascular invasion (3). RC is not an option for intermediate-risk tumors.

EAU, NCCN, or AUA guidelines do not mention the specific treatment of a low-grade, extensive Ta tumor, not exposed to BCG treatment, and not amenable to endoscopic resection. The AUA guideline states that for a Ta low- or intermediate-risk patient, the clinician should not perform an RC until bladder-sparing modalities (staged TURBT, intravesical therapies) fail. We only found one reference (case series) supporting this statement for RC in such tumors (25).

Although a multifocal or very large LG NMIBC represents a rare situation, RC might be effective and considered in these cases where repeated endoscopic resections fail to succeed (Figure 2c, 2d and 2e).

## DISCUSSION

Non-muscle invasive bladder cancer (NMIBC) is a disease that can commonly be cured through transurethral resection of the bladder tumor (TURBT). The high recurrence rates and progression risk are avoided through adjuvant measures and an extensive follow-up program (3).

Although TURBT is a standard procedure mastered by most urologists, there are certain challenging situations to discuss. Sometimes urologists face an unreachable NMIBC, a high-volume prostate, or a urethral stricture that precludes the resectoscope introduction, or an extensive low-grade Ta tumor that cannot be endoscopically resected.

In this review, we have found some studies helping to have some insights on how to manage these patients, although those are low-quality evidence. Very distinctive strategies have been reported in the literature, mainly descriptive, anecdotally, or small randomized studies.

**Figure 3:**
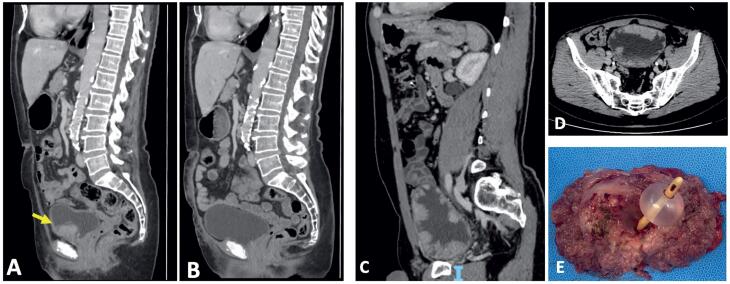
Computed Tomography (CT) scan of a patient who underwent a partial cystectomy for an anterior bladder neck LG-NMIBC (yellow arrow) could not be adequately resected endoscopically. A) preoperative image. B) After 22 months of follow-up. Radical cystectomy was performed for an extensive LG Ta NMIBC. C) and D) CT scans demonstrating extensive LG-Ta lesion. E) surgical aspect of the bladder after radical cystectomy.

Some of these treatments outlined in the present manuscript include repeated TURBTs, chemoablation, BCG immunoablation, partial cystectomy, radical cystectomy, radiotherapy, and chemotherapy. In summary, we have combined these strategies into a proposed algorithm to be considered in this situation (Figure-3).

Another vital consideration for decision-making is prognostic factors and how to improve the outcomes with a more invasive procedure. Regarding the first one, we need to identify high-risk recurrence and progression patients with algorithms, artificial intelligence, or laboratory tools. To predict oncological outcomes and optimal, tailored therapeutic decision-making, we have found that a high neutrophil-to lymphocyte ratio (NLR) was already consistently associated with locally advanced disease. Also, it represents an independent prognostic factor of recurrence and progression in NMIBC patients (26). For the second issue, the Enhanced recovery after surgery (ERAS) program has been described as an alternative to reduce the perioperative morbidity and mortality in patients undergoing a radical cystectomy (27, 28). Therefore, we may counsel every urologist to follow these recommendations when deciding to perform an RC in these settings.

From a future perspective, the landscape of new drugs for the treatment of bladder cancer has widely improved in the last decade. The pathophysiology knowledge and genomic profile of such tumors have also been increasing rapidly (29). In such a context, we might have a near-future further option for these uncommon situations of challenging LG-Ta NMIBC. Immuno-oncology and targeted therapies have already been used for specific situations of NMIBC.

Some new drug trials evaluate oncolytic virus regimen, recombinant fusion proteins, immune modulation, cytotoxic therapies, and targeted small molecule kinase inhibitors. As research improves, we are likely to see an increase in the number of options for such patients.

## CONCLUSIONS

For those patients with an unreachable LG-NMIBC, a high-volume prostate, an urethral stricture that precludes the resectoscope introduction, or an extensive low-grade Ta tumor, we have found that repeated TURBTs, chemoablation, BCG immunoablation, partial cystectomy, radical cystectomy, radiotherapy, and chemotherapy are attractive modalities to treat them effectively. Also, the current manuscript proposes an algorithm to overcome these challenges. We also consider that there is a wide gap to fill in with high-quality evidence.
